# Robotic System for Physical Training of Older Adults

**DOI:** 10.1007/s12369-020-00697-y

**Published:** 2020-09-30

**Authors:** Omri Avioz-Sarig, Samuel Olatunji, Vardit Sarne-Fleischmann, Yael Edan

**Affiliations:** Department of Industrial Engineering and Management, Ben-Gurion University of the Negev, Beer-Sheva, 8410501 Israel

**Keywords:** Older adults, Physical activity, Feedback, Humanoid robot, Kinect, NAO, Poppy

## Abstract

Physical exercise has many physical, psychological and social health benefits leading to improved life quality. This paper presents a robotic system developed as a personal coach for older adults aiming to motivate older adults to participate in physical activities. The robot instructs the participants, demonstrates the exercises and provides real-time corrective and positive feedback according to the participant’s performance as monitored by an RGB-D camera. Two robotic systems based on two different humanoid robots (Nao, toy-like and Poppy, mechanical-like) were developed and implemented using the Python programming language. Experimental studies with 32 older adults were conducted, to determine the preferable mode and timing of the feedback provided to the user to accommodate user preferences, motivate the users and improve their interaction with the system. Additionally, user preferences with regards to the two different humanoid robots used were explored. The results revealed that the system motivated the older adults to engage more in physical exercises. The type and timing of feedback influenced this engagement. Most of these older adults also perceived the system as very useful, easy to use, had a positive attitude towards the system and noted their intention to use it. Most users preferred the more mechanical looking robot (Poppy) over the toy-like robot (Nao).

## Introduction

The aging population rate is rising rapidly [1]. With age there is a decline of physical and mental capability [2, 3]. Inactivity of older adults often results in functional decline, loss of independence and increased disease burden [4]. Physical activity via exercises can delay, prevent, or even reverse these effects [4]. However, older adults do not engage in exercises as much as is recommended for their health [4].

Several technologies have been developed to encourage physical activities such as exergames for mobility [5] (e.g. Wii Fit [6, 7]), virtual reality simulators [8], smartphone applications [9], embodied conversational agents, video-based games and dance [5]. Robot coaches have been used to encourage children exercising [10] and have been proved to be effective in terms of companionship and social interaction [10]. Previous research revealed that robot coaches are as effective as a human coach [11] and preferable to a virtual coach [12–14].

Development of robotic applications in the eldercare domain [15] have been aimed at providing physical support^1^, ^2^ [16], social interaction^3^ [17–20], cognitive stimulation [18, 21] and safety monitoring^4^ [22]. The current work focused on development of a robotic system for physical training of older adults. Interaction parameters related to the feedback provided by the robot as well as the influence of the appearance of the robot were evaluated.

Feedback, as an important element of the interaction with the robot, must be provided appropriately to be effective [23–25]. Positive feedback during exercise sessions significantly benefits the experience of older adults [12, 26]. Negative feedback is known to reduce the user’s motivation [27, 28].

Feedback can be provided to the users via different modalities, including: tactile, haptic, auditory, visual or any combination of those [29–32]. Speech is considered a natural means of providing feedback, and can provide encouragement and support to increase the users motivation [32]. However, it may also distract the user, and might be missed in the case of elderly people who may suffer from a decreased hearing [33]. Visual feedback suitable for remote human–robot collaboration [29] could include LEDs to provide better understanding of the robot’s state and actions [34]. Facial expression is an important mean of visual feedback in HRI [35], since it can provide useful information [35]. It also has the potential to make the robots behavior more understandable [36] and attractive [25] since the robot seems more intelligent. Combination of visual feedback and speech can be efficient [32, 35] and lead to improved collaboration between the human and the robot [34].

The timing [37] of feedback influences the interaction with the system and its effectiveness [32, 34]. Feedback can be immediate or delayed, frequent or infrequent [23]. Suitable timing of feedback advances natural flow of communication which helps the user accept the robotic agent communication partner [37]. Adequately timed feedback maintains comprehension in the communication, whereas feedback given too late causes confusion. The temporal proximity between users’ input and the robots’ response also has been noted to affect the naturalness of the interaction [38]. Timing of feedback could also be continuous or discrete. Continuous feedback proved to improve the trust of the user in the robot [39, 40] and decrease the users’ workload [39]. However, continuous feedback could lead to presentation of too much information [41]. This can result in information overload and decrease the performance of the user [41].

The current study aimed to find the suitable mode and timing of the feedback provided by a physical training robot for older adults, in order to improve the interaction between the users and the robot as well as to increase the users’ motivation to consistently engage in their exercise sessions.

Previous research also revealed that robots with anthropomorphic appearance tend to engage users to interact with the robots in a way that is similar to human-human interaction [42]. This tuned the ongoing development of robotic coaches towards the direction of humanoid robots or robots with some semblance of human-likeness [12]. Such developments involving humanoid robot coaches [12, 13] engaged the users in upper limb training sessions. The robots (Bandit and R1) were larger in dimension, closer to human-size and mounted on a wheeled base. This presents a different anthropomorphic impression on the user compared to the smaller sized humanoid robots used in our study which we presume can be more easily handled by the user for personal use. The robots used in our study also had higher degrees of freedom compared to Bandit, providing a wider range of exercises.

We therefore also conducted an exploration of user preferences while training with two forms of humanoid robots to determine which of the robots yields better satisfaction.

The article is organized as follows: Sect. 2 presents details of the overall system design while Sect. 3 describes the method used for experimental evaluations of the design. Section 4 is devoted to the results of the experiments conducted followed by Sect. 5 that discusses the obtained results. Conclusions and suggestions for future work are summarized in Sect. 6.

## System Design

### Overview

The system design was focused on motivating older adults to regularly observe and complete their exercise sessions. It was hypothesized that system effectiveness depends on the robot type and on the timing and mode of feedback which would encourage the users to cooperate and complete the training. The older adults may not be aware if their actions accurately corresponded to what the robot required them to do. The robot therefore notified them if they were not doing the exercises correctly. This ensured that the older adults were aware of the correctness of their actions at every point of time [39]. The most suitable timing and mode of feedback for improved interaction were therefore explored.

**Fig. 1:**
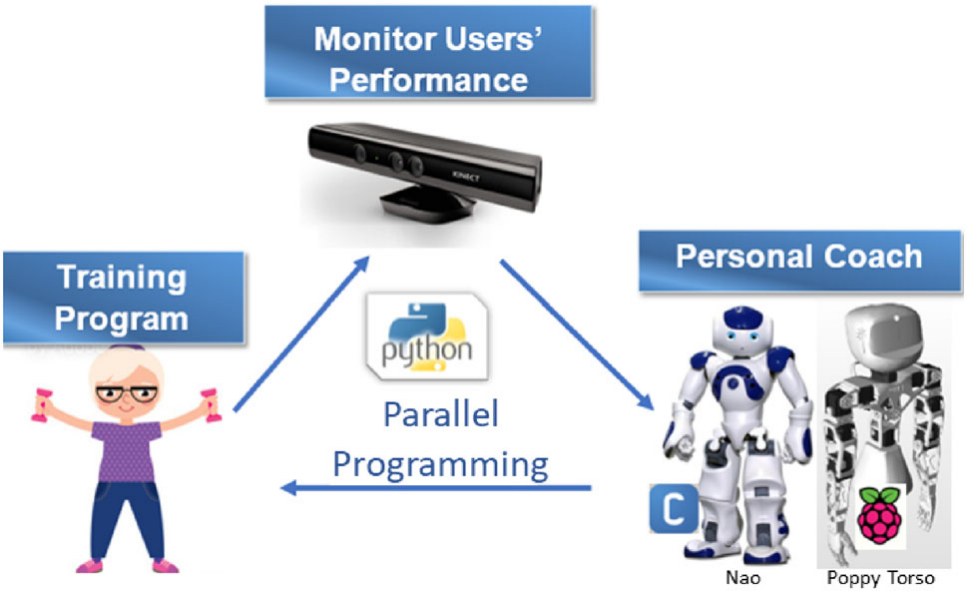
The Developed System

The overall description of the system components is described in the following subsections:

### Hardware Description

The system hardware included a humanoid robot (Fig. 1), which demonstrates the exercises for the users, connected to a RGB-D camera to monitor the users performance and exercise sessions.

Two types of humanoid robots were used as robotic coaches for comparison purposes (Fig. 1): Nao robot (a toy-like robot) and Poppy robot (a more mechanical-looking robot).

The Kinect was used as the RGB-D camera for tracking performance because it was accessible, applicable and had a software development kit equipped with skeleton tracking. The depth camera provides a more accurate view of the users movements compared to a conventional RGB camera. This aids the feedback process by giving the users information regarding their performance. The robotic coach was programmed to instruct the users for the different forms of exercises.

The developed algorithm included parallel programming of each robot with the Kinect.

#### Nao Robot

Nao is an autonomous, programmable humanoid robot developed by Aldebaran-Robotics, a French robotics company^5^. Its height is 57 cm and its weight is 4.3 kg [43]. Nao’s main technical features include 25 degrees of freedom (DOF), sensors in its head, hands and feet, sonars, 4 directional microphones and loudspeakers, and two cameras. The operating system is NAOqi which is an embedded GNU/Linux distribution.

#### Poppy Robot

Poppy is an open-source 3D printed humanoid robot. Its full body height and weight are 53cm and 3.5 kg, respectively [44]. Only the torso of the robot was available at the time of the study and it was therefore used in the experiments without the legs. Poppy was designed to be anthropomorphic with 25 DOFs including a 5 DOFs articulated trunk [45] and LCD screen for display, on which a facial impression was added.

#### Kinect

The Microsoft Kinect sensor for windows is a low-cost sensor that integrates a depth sensor, color camera, and a microphone [46, 47]. The sensor can provide full-body 3D motion capture, skeleton data, voice recognition and facial recognition [47].

### System Development

System development included characterization and development of the exercises, monitoring of the users’ performance, and programming of the operations of the robot coach.

#### Development of the Exercises

The developed training program included 8 strength exercises which were part of those recommended by the National Institute on Aging (NIA).^6^ These exercises were chosen because muscle weakness is a widespread problem among older adults. This leads to difficulty and inability to perform basic activities such as getting out of bed or from the chair, opening a bottle, and carrying objects. It is also a high risk factor for falls among older adults [48]. Muscle strengthening can help making daily activities easier and hence these exercises are recommended for older adults [49].

**Fig. 2:**
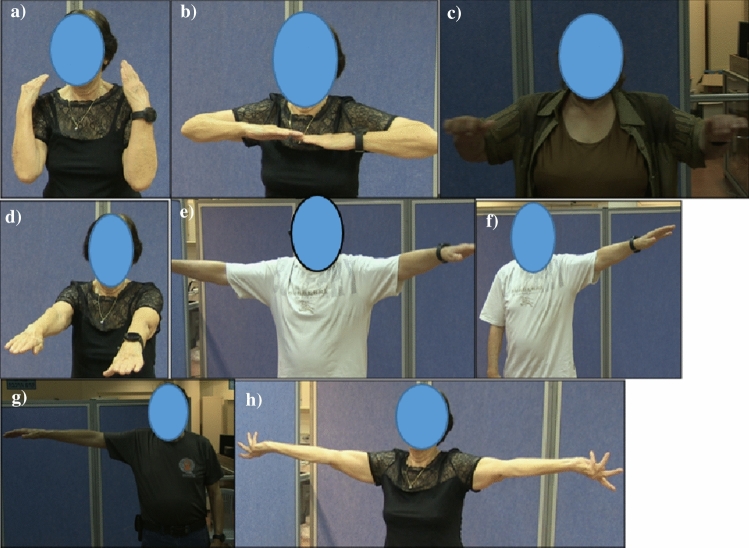
**a** bending elbows, **b** raising arms and bending elbows, **c** raising arms and bending elbows 90 degrees, **d** raising arms forward, **e** raising arms horizontally, **f** raising left arm horizontally, **g** raising right arm horizontally, **h** raising arms horizontally and turning hands

The following strength exercises were implemented: bending elbows, raising arms and bending elbows, raising arms and bending elbows 90 degrees, raising arms forward, raising arms horizontally, raising left arm horizontally, raising right arm horizontally, and raising arms horizontally and turning hands. The illustrations for the exercises are presented in Fig. 2.

#### Monitoring Users Performance

Users performance was monitored with the Kinect camera which was programmed to extract the users’ skeleton. The skeleton contained the 3D point position of 25 joints of the user. Each position included a coordinate system (*X*, *Y*, *Z*). *X* corresponded to the width, *Y* corresponded to the height and *Z* to the depth.

1 $$\begin{aligned} d = \sqrt{(x_2-x_1)^2+(y_2-y_1)^2} \end{aligned}$$

2 $$\begin{aligned} \cos \beta = \frac{a^2+b^2-c^2}{2ab} \end{aligned}$$

**Fig. 3:**
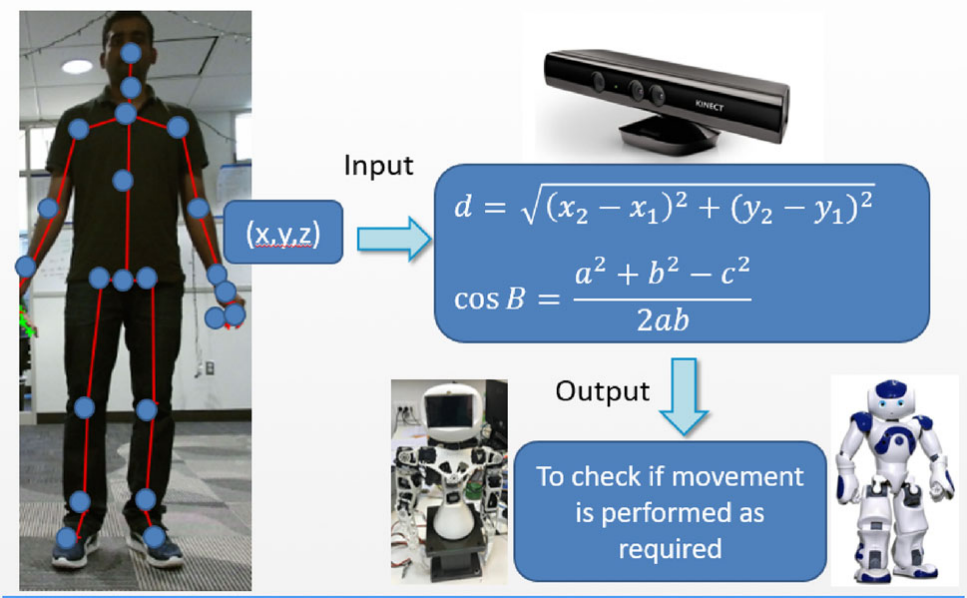
Kinect’s skeletal tracking in real time

The distances and the angles between joints were calculated, as presented in Eqs. (1) and (2) respectively. The following algorithm was developed to check if the user made the correct movement (Algorithm 1). The image of the Kinect’s skeletal tracking in real time is also depicted in Fig. 3.

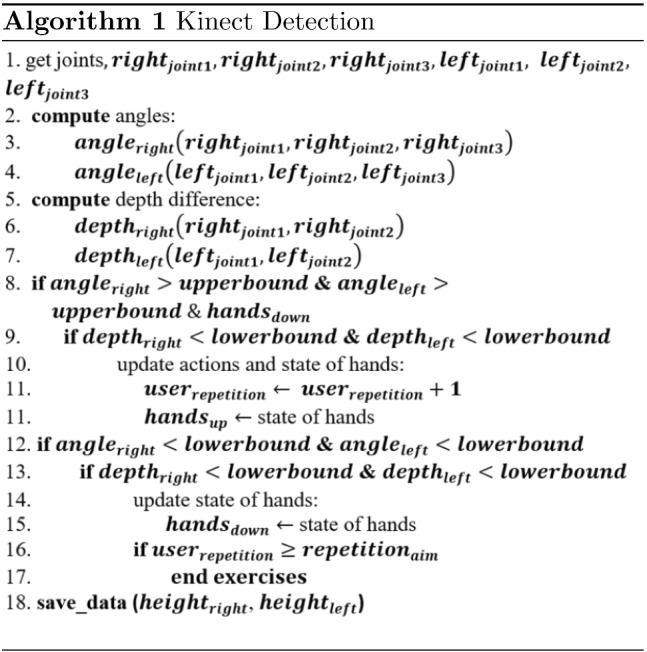



#### Operations of the Robot Coach

**Fig. 4:**
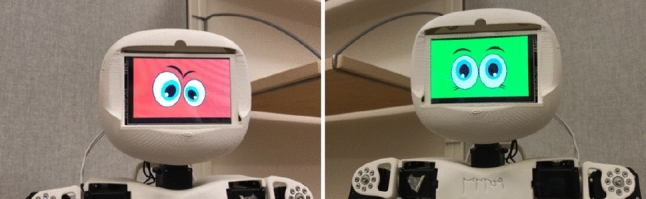
Visual feedback of Poppy Robot

The two robot coaches (Nao and Poppy) were programmed with the Python programming language. The Nao robot was programmed using the *naoqi* library while the Poppy robot was programmed with the *pypot* library. The robots’ speech was implemented with existing algorithms in these libraries. This was chosen based on previous research that indicated that human voice is more socially acceptable as compared to synthetic robotic voices [50]. A screen running on raspberry pi was added to the Poppy robot to provide visual (facial) feedback as shown in Fig. 4. It was assumed this would engage the older adults more as noted in previous research regarding feedback and humanoid facial design [25, 35, 36, 51].

**Fig. 5:**
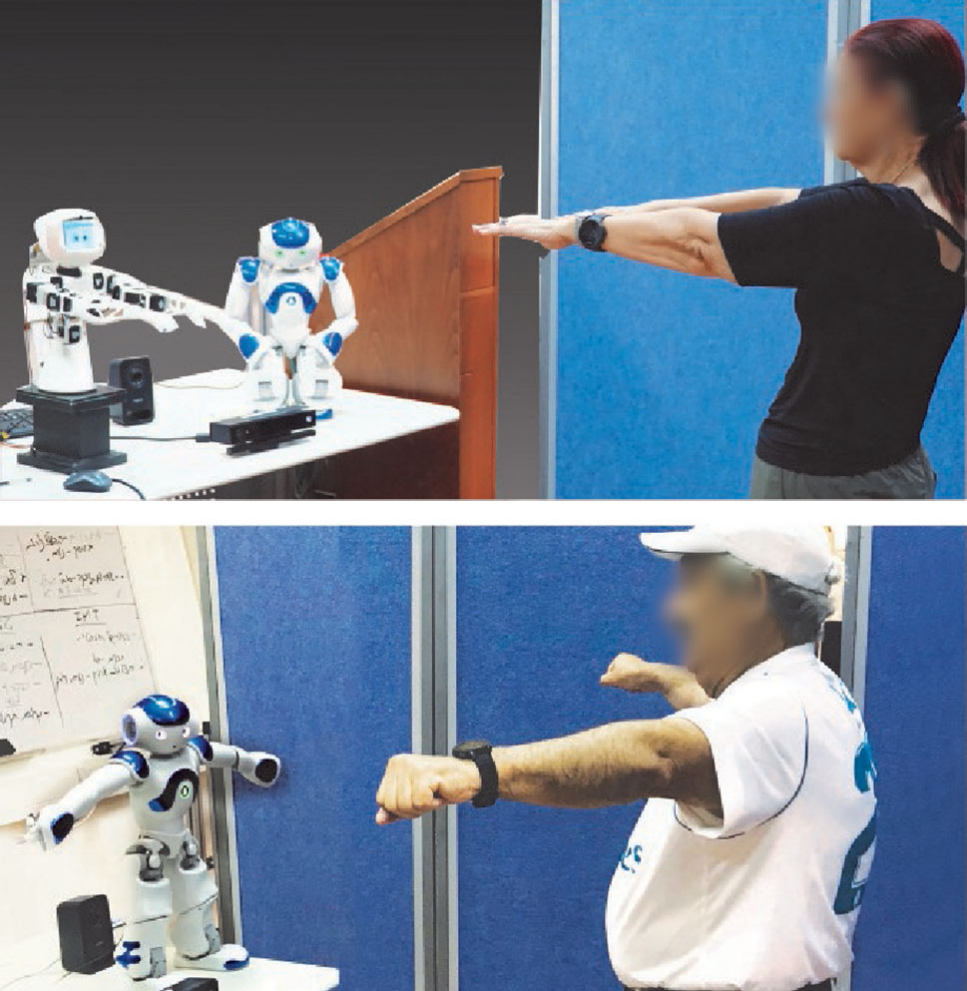
Robot demonstrating exercise for participants

**Fig. 6:**
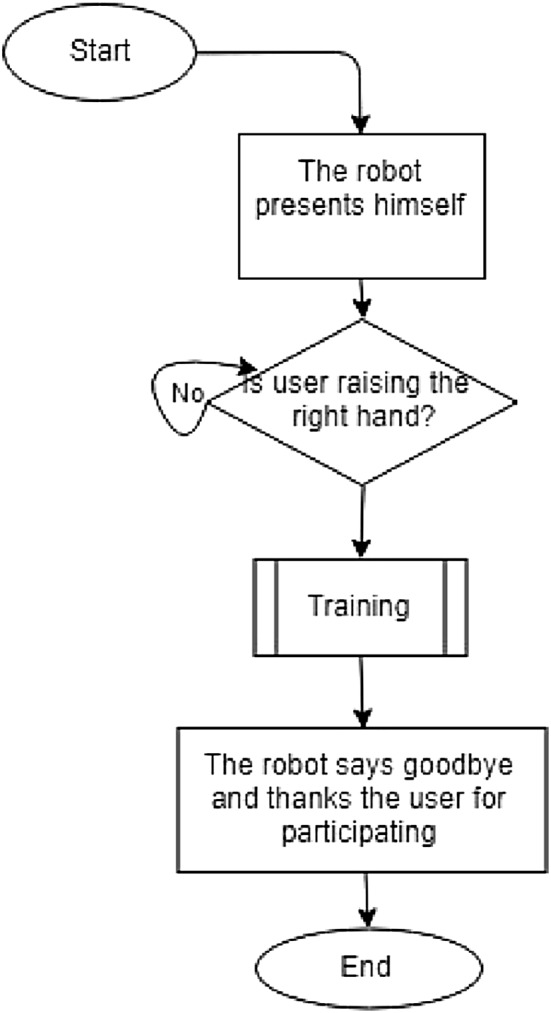
Main Function

**Fig. 7:**
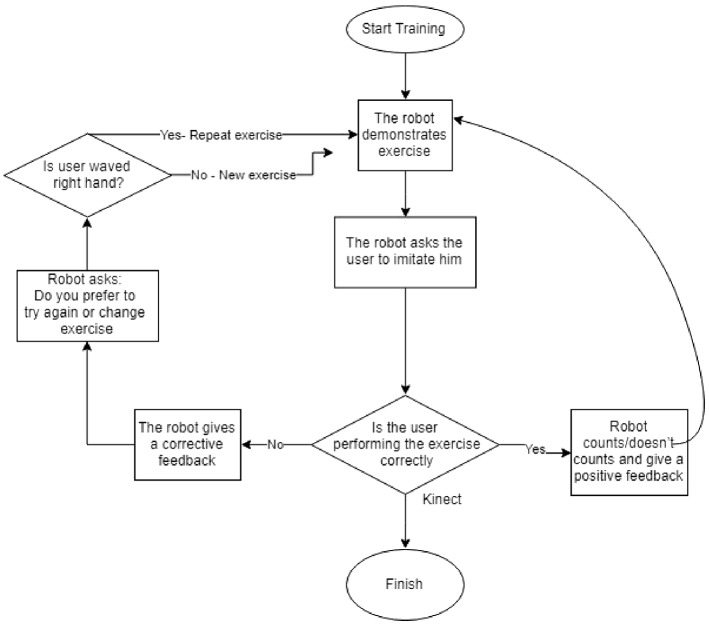
Strength Exercises

The session began with the robot standing in front of the user. The robot waited until the user stood in front of it (Fig. 5). It introduced itself as the users’ exercise coach. It asked the user to raise his right hand if he/she wanted to start the training. Then, the robot started the session with instructions on what exercise should be done and also demonstrated how it should be done. At the end of the session, the robot thanked the user for his/her participation and said goodbye (Fig. 6). The process for the exercises is given in Fig. 7 and can be seen in the video: https://smartrobabcbgu.wixsite.com/iemirl/interactive-robotics-research-netwo.

## Method

### Model

**Fig. 8:**
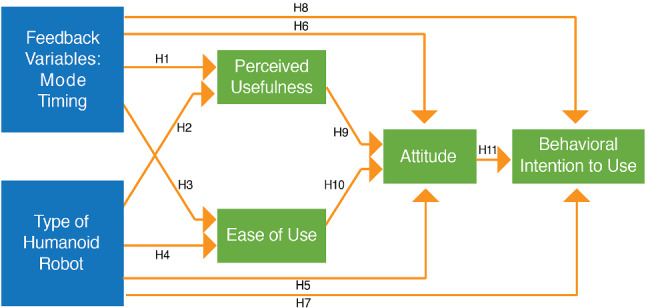
The study model (based on the Technology Acceptance Model [52])

The model for the study was designed based on the *“Technology Acceptance Model”* [52]. The independent variables in the experiment were the mode and timing of feedback and type of robot. Although both Poppy and Nao are humanoid robots, Poppy was hypothesized to be more likable due to the facial impression that was added using the LCD screen and due to its appearance which is less toy-like. This conforms with the study of the design and perception of humanoid robot headed by DiSalvo et al. [51] where they emphasized the significance of well expressed robot eyes in the human’s perception of the robot. Additionally, Poppy’s movements reach a wider range than the Nao and therefore its demonstration of exercises might look to the users more real. The dependent variables were the perceived usefulness, ease of use, attitude and behavioral intention to use the system as depicted in Fig. 8. These were measured objectively and subjectively. The following hypotheses were proposed:

- **H1** - *H1a*: Older adults will perceive the system as more useful in conditions with audio and visual feedback compared to conditions when only audio feedback is present. *H1b*: Older adults will perceive the system as more useful with continuous feedback compared to discrete feedback.
- **H2** - Older adults will perceive the system as more useful while interacting with Poppy robot compared to the Nao robot.
- **H3** - *H3a*: Older adults will perceive the system as easier to use when receiving audio and visual feedback compared to only audio feedback. *H3b*: Older adults will perceive the system as easier to use with continuous feedback compared to discrete feedback.
- **H4** - Older adults will perceive the system as easier to use when interacting with Poppy robot (mechanical like) compared to Nao robot (toy-like).
- **H5** - Older adults will have a better positive attitude while training with Poppy robot than with Nao robot.
- **H6** - *H6a*: Older adults will have a more positive attitude while using the system with audio and visual feedback than with only audio feedback. *H6b* Older adults will have a more positive attitude while using the system with continuous feedback than with discrete feedback.
- **H7** - The behavioral intention to use the system of the older adults will be higher with Poppy robot than with Nao robot.
- **H8** - *H8a*: The behavioral intention to use the system of the older adults will be higher with audio and visual feedback than with only audio feedback. *H8b* The behavioral intention to use the system of the older adults will be higher with continuous feedback than with discrete feedback.
- **H9** - Higher perceived usefulness of the system will influence a positive attitude to use the system.
- **H10** - Higher ease of use of the system will influence a positive attitude to use the system.
- **H11** - Positive attitude will influence higher behavioral intention to use the system.

### Experimental Design

A between and within participants experimental design was conducted with three independent variables (Table 1): mode of feedback (audio and a combination of audio and visual), timing of feedback (discrete and continuous) and type of robot (Poppy-mechanical looking and Nao-toy like). The participants were distributed in 4 groups. Each participant experienced one combination of mode and timing of feedback (between-subjects) with both of the humanoids robots, Poppy and Nao, (within-subjects).

Audio feedback was provided as a recorded non-synthesized female voice. This was identical for both robots. For the discrete mode of feedback, a spoken message approving or disapproving the users performance at the end of each exercise was used. For the continuous mode, a counting of the repetition of movements during the exercises was used, in addition to the discrete feedback provided. Visual feedback was implemented only for the discrete mode and was provided differently for each robot according to its capabilities. For the Nao robot, visual feedback was provided using its LED lights (green or red) on its eyes and body. For the Poppy robot visual feedback was provided by presenting a positive green face or a negative red one on the LCD screen (as can be shown in Fig. 4). The order of presentation of robots (Nao-Poppy, Poppy-Nao) was randomized to account for potential bias.

**Table 1:** Experimental design

Mode / Timing	Discrete	Continuous
Audio	Poppy	Nao
	Nao	Poppy
Audio and visual	Nao	Poppy
	Poppy	Nao

### Experimental Procedure

The experiment included two sessions where participants interacted with a different robot in each session. In each session the user was engaged with the robot to carry out the exercises demonstrated by the robot. The user was prompted by the robot to imitate it. There was an introduction session by the robot as described in Fig. 6. Then, the robot proceeded to the actual strength exercises. The exercises were monitored by the Kinect camera to ensure compliance with the robots’ instructions.

### Participants

Thirty two healthy older adults were recruited by snowball sampling which started through advertisements at the following sources: Ben-Gurion University’s Center of Digital Innovation healthy aging innovation lab,^7^ an older adults local club in Beer Sheva, a local police pensioners club, BGU’s older adults working force and older adults who participated in former experiments that took place in our labs.

The participants were composed of 18 females and 14 males aged between 70 and 88 ($M\,{=}\,77.4$, *SD* = 5.8). The educational level of 6.3% of the participants was Ph.D., 15.6% had a masters degree, 28.1% had a bachelors degree, 31.3% had a secondary education and 18.8% had other education. All recruited participants contacted the authors and filled the consent form independently.

### Evaluation

The dependent variables were assessed objectively and subjectively.

#### Objective Performance Measures

A compilation of the objective measures taken during the study are presented in Table 2.

Comfort of the participants was considered while assessing the ease of use of the system. Comfort was measured through the heart rate (HR) readings of the participants during the physical exercises [53, 54]. It also reflected, to some extent, the physical demand placed on the participants [55]. These measurements were acquired from a Garmin watch worn by the participants along the experiments (Forerunner 235 series).^8^ Heart rate change (HR change) was calculated based on the heart rate readings before and after the exercise session as presented in Eq. (3).

3 $$\begin{aligned} HR\ Change = \frac{HR\ after - HR\ before}{HR\ before} \end{aligned}$$

The following measures were acquired from the video recordings of the sessions. Understanding was measured in terms of the time (seconds) it took participants to react to the robot’s instructions. This could be an indication of how much understanding the participant had regarding the instruction the robot gave. Another objective measure of understanding taken were observations of participants who continued with the exercise after the robot had stopped. In some instances, the robot completed the exercise before the participants. Understanding the robots’ instructions and feedback implies that the participant continues and completes the number of repetitions instructed by the robot even after the robot has completed its own rounds. We used this as a measure of assessing how well the participants comprehended the instructions and feedback given by the robot.

The attitudes of the participants in terms of engagement and adherence to training were objectively assessed as eye-contact duration and success rate (SR) respectively. The eye-contact duration was determined by watching the videos of the trials after the experiments and calculating the ratio of the participants’ “no eye-contact time” to trials total time. The success rate of the participants in the exercises was computed based on the number of exercises completed in the total number of exercises as shown in Equation (4).

4 $$\begin{aligned} SR = \frac{exercises\ completed}{sum\ of\ exercises} \end{aligned}$$

**Table 2:** Objective measures

Dependent variable		Measurement
Ease of use	Comfort	heart rate change [53, 54]
	Understanding	Reaction time (s) [56]
		Participant continued with the exercise after the robot had stopped
Attitude	Engagement	Eye contact (*duration* $of \ gazes \ at \ the \ robot)$ [57]
	Adherence to training	Success rate

#### Subjective Performance Measures

The subjective measures were collected through questionnaires which involved questions about the participants’ experience with the robot, technology acceptance and negative attitude towards robots. The pre-trial questionnaire used was composed of demographics questions for the participants, the Negative Attitudes Towards Robots Scale (NARS) [58], and Technology Assessment Propensity (TAP) [59]. NARS questionnaire examines the participants perception of technology and robots while TAPs questionnaire examines the participants level of technological knowledge. The participants responded to the questionnaires on a 5-point Likert scale ranging from “1 = strongly disagree” to “5 = strongly agree”. The post trial questionnaire and the variables it assessed are presented in Table 3. The questionnaire was based on the *Almere model* [60]. The participants indicated their level of agreement on a 3-point Likert scale ranging from “1 = disagree” to “3 = agree”. The 3-point Likert scale was used in the post-trial questionnaire in order to avoid the mental workload some of participants reported in other studies [61] that they experienced while trying to rate their experience on questionnaires of higher scales.

**Table 3:** Post trial questionnaire

Dependent variable		Question
Perceived usefulness		I would be willing to train with the robot again because it had value to me
Ease of use	Comfort	I felt nervous during the activity
		felt comfortable during the interaction
	Understanding	I understood the robot well during the interaction
	Effort	I put a lot of effort into this activity
Attitude	Engagement	I concentrated on the activity for the entire session
	Trust	I felt like I could really trust this robot
	Satisfaction	I was satisfied by the robot’s performance during this activity
	Enjoyment	I enjoyed the activity
Intention to Use	Acceptance	I would like to exercise with the robot in the future

The final questionnaire included questions regarding the evaluation of the robot as exercise coach (Table 4).

**Table 4:** Final Questionnaire

1. Should the robot count?	
2. Which robot did you understand better?	
3. Which system would you choose to use?	
4. Would you like to train with the system in the future?	

#### Analyses

The analysis and the pre-processing of the raw data was conducted with the following statistical programs: *SPSS, RStudio, Excel*. The influence of the independent variables on the objective dependent variables were evaluated using the generalized linear mixed model (GLMM). The target function was chosen according to the distribution of the independent variable.

## Results

### Participants Characteristics

The characteristics of the participants analyzed based on pre-questionnaires they filled is described in this subsection. This is analyzed in relation to their responses to the TAP and NARS questionnaires. This was done to observe the overall attitude of the participants to adopting new technology and any predisposition they had towards robot before interacting with the physical training robots. The influence of gender was not compared since there was a significant difference in both age and educational level distributions of the participants which might bias the results.

#### TAP—Technology Adoption Propensity

The responses of the participants to the TAP questionnaire [59] revealed that 3.23% of the participants had a low propensity to adopt technology, while 38.71% and 58.06% had medium and high propensity adoption levels respectively (*M* = 3.54, *SD* = 0.65).

80.65% of the participants strongly believed that technology provides increased control and flexibility in life. 41.94% of the participants had a low confidence in their ability to quickly and easily learn to use innovative technologies, as well as a sense of being technological. 32.26% of the participants had high confidence in such ability, the remaining 25.81% were indifferent about it. 58.06% of the participants strongly thought that they were overly dependent on technology, and had a feeling of being enslaved by it, while 35.48% were neutral about it.

**Fig. 9:**
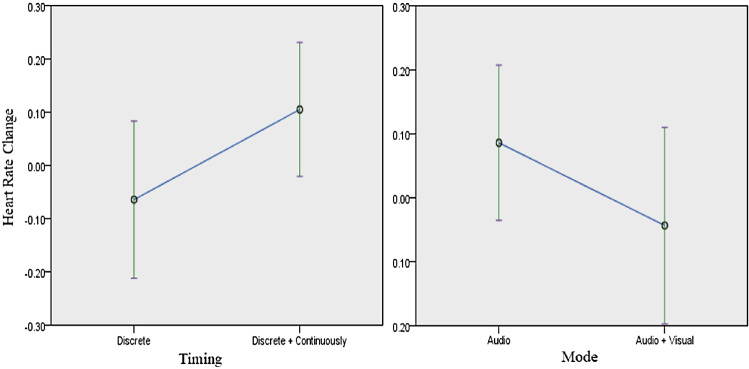
Heart Rate Change as influenced by Timing (left) and Mode (right) of Feedback

#### NARS—Negative Attitude Towards Robots Scale analysis

The NARS results revealed that 35.48% of the participants had a negative attitude towards situations and interactions with robots while 51.61% were neutral about it. 22.58% had highly negative attitudes towards the social influence of robots, 51.61% had a low attitude and 25.81% were neutral about it. 64.52% had a highly negative attitude towards the concept of robots having emotions, 9.68% were indifferent about it while 25.81% had a low negative attitude towards it.

### Evaluation of the Training

#### Participants’ Interaction

The results of the interaction of the participants with the robot coach for the physical training is described in this subsection. The influence of the main variables tested as the participant interact with the robot are presented in terms of the aforementioned dependent measures.

#### Perceived Usefulness

Majority of the participants indicated through the questionnaire that they perceived the robot as useful for them. More of the participants in the audio feedback group (77.4%) indicated their willingness to train with the robot in the future compared to participants who experienced both audio and visual feedback (66.7%) (contrary to *H1a*). More of the participants in the continuous feedback group (77.8%) indicated their willingness to train with the robot in the future compared to participants who experienced discrete feedback (64.3%) (in line with *H1b*). The robot type did not influence the perceived usefulness—participants rated both robots equally - 71.9% (contradicts *H2*).

#### Ease of Use

The ease of use of the system, as assessed by the change in heart rate, was significantly affected by the mode and timing of the feedback, but not by the robot type. Details of the influence of these independent variables on the heart rate are as follows: in terms of the mode of feedback ($F(1,60) = 4.101$, $p = 0.047$), participants with audio feedback (*M* = 0.23, *SD* = 0.31) had higher change in heart rate than participants with combined audio and visual feedback (*M* = 0.17, *SD* = 0.23) (confirming *H3a*). With regards to the timing of feedback ($F(1,60) = 7.674$, $p = 0.007$), the heart rate change of participants with continuous feedback (*M* = 0.26, *SD* = 0.29) was higher than that of the participants with discrete feedback (*M* = 0.13, *SD* = 0.23) (contradicting *H3b*). This is portrayed in (Fig. 9). Exercising with Nao or Poppy robot did not have a significant difference on the heart rate ($F(1,62) = 0.318$, $\rho$ = 0.575) (contrary to *H4*).

**Fig. 10:**
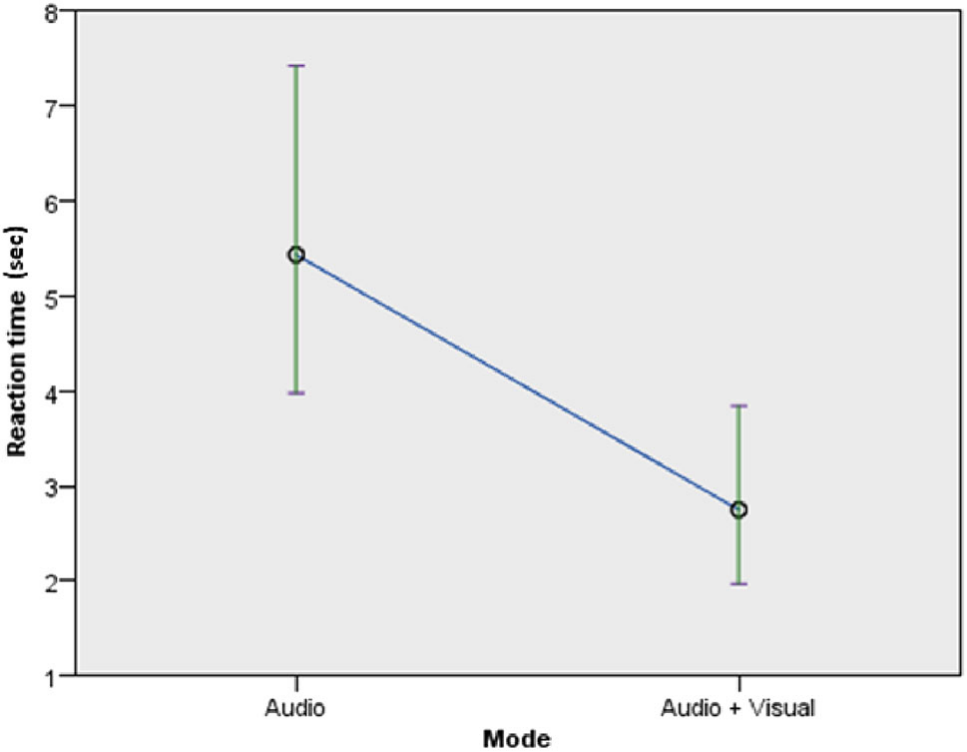
Reaction time by mode of feedback

The reaction time (in s) of the participants was significantly affected by the mode of feedback but not by the timing of feedback and robot type. Details regarding the influence of the mode of feedback ($F(1,58) = 8.931$, *p* = 0.004) (Fig. 10) revealed that there was a reduction in the reaction time of those using both audio and visual feedback (*M* = 2.7, *SD* = 1.845) as compared to participants with only audio feedback (*M* = 5.45, *SD* = 7.82) (confirming *H3a*).

In terms of the timing of feedback ($F(1, 58) = 0.704$, *p* = 0.405), there was a reduction in the reaction time for participants who experienced continuous feedback (*M* = 3.53, *SD* = 5.165) as compared to participants with discrete feedback (*M* = 4.54, *SD* = 6.21) (in line with *H3b*). With regards to the robot type ($F(1,60) = 0.011$, $\rho$ = 0.918), the reaction time of participants that used the Poppy robot (*M* = 3.9, *SD* = 6.05) was shorter than participants who used the Nao robot (*M* = 4.06, *SD* = 5.23) (in line with *H4*).

**Fig. 11:**
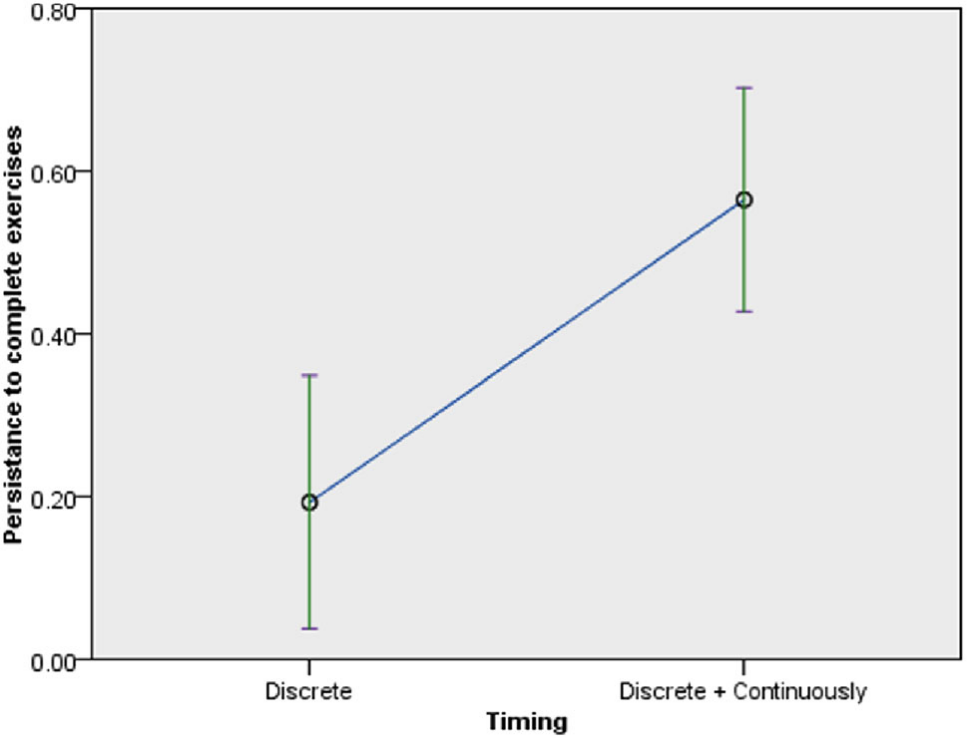
Persistence to complete exercise by timing of feedback

Regarding the persistence to complete exercises, timing of feedback had significant influence but the mode of feedback and robot type did not have significant influences. Details of the influences on the persistence to complete exercises revealed the following: in terms of the timing of feedback ($F(1,60) = 12.822$, *p* = 0.001) (Fig. 11), the participants persistence to complete the exercises with continuous feedback (*M* = 0.57, *SD* = 0.45) was higher than the persistence with discrete feedback (*M* = 0.18, *SD* = 0.37) (confirming *H3b*). In terms of the mode of feedback ($F(1, 60)=2.697$, *p* = 0.106), audio and visual feedback (*M* = 0.5, *SD* = 0.47) influenced higher persistence than audio feedback (*M* = 0.3, *SD* = 0.41) (in line with *H3a*). In terms of the robot type ($F(1,62) = 0.677$, *p* = 0.414), the persistence of participants with the Poppy robot (*M* = 0.35, *SD* = 0.45) was lower than that of participants who used the Nao robot (*M* = 0.45, *SD* = 0.46) (contrary to *H4*).

Almost all the participants indicated in the questionnaire that they understood the robot well during the interaction. Regarding the feedback mode, higher percentage in the audio feedback group (96.8%) indicated that they understood the system better compared to participants who experienced the combined audio and visual feedback (87.9%) (contrary to *H3a*). Regarding the feedback timing, all the participants in the continuous feedback group indicated that they were comfortable with the system while 82.1% of the participants who experienced discrete feedback indicated that they were comfortable (in line with *H3b*). There was only a slight difference in the robot type preference regarding the comfort—90.6% preferred Poppy while 93.8% preferred Nao (contrary to *H4*).

Most participants noted that the activity did not require much effort from them. The participants in the audio and visual feedback group (78.8%) reported that the training required less effort compared to participants who experienced only the audio feedback (67.7%) (in line with *H3a*). Regarding the timing of feedback, the participants in the continuous feedback group (80.6%) reported that they exerted less effort compared to participants who experienced discrete feedback (64.3%) (in line with *H3b*). There was only a slight difference in the robot type preference regarding the effort, 75% preferred Poppy while 71.9% preferred Nao (in line with *H4*). The participants had the opportunity to select both robots if they liked both robots or none if they liked neither of them. These percentages, therfore, do not add up to 100% because some of the participants selected both robots.

#### Attitude

**Fig. 12:**
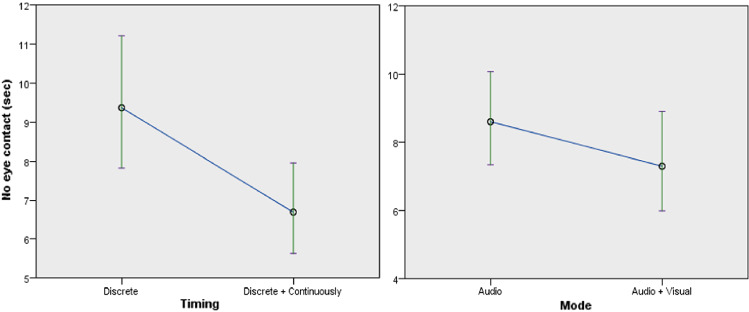
No eye contact by timing and mode of feedback

**Fig. 13:**
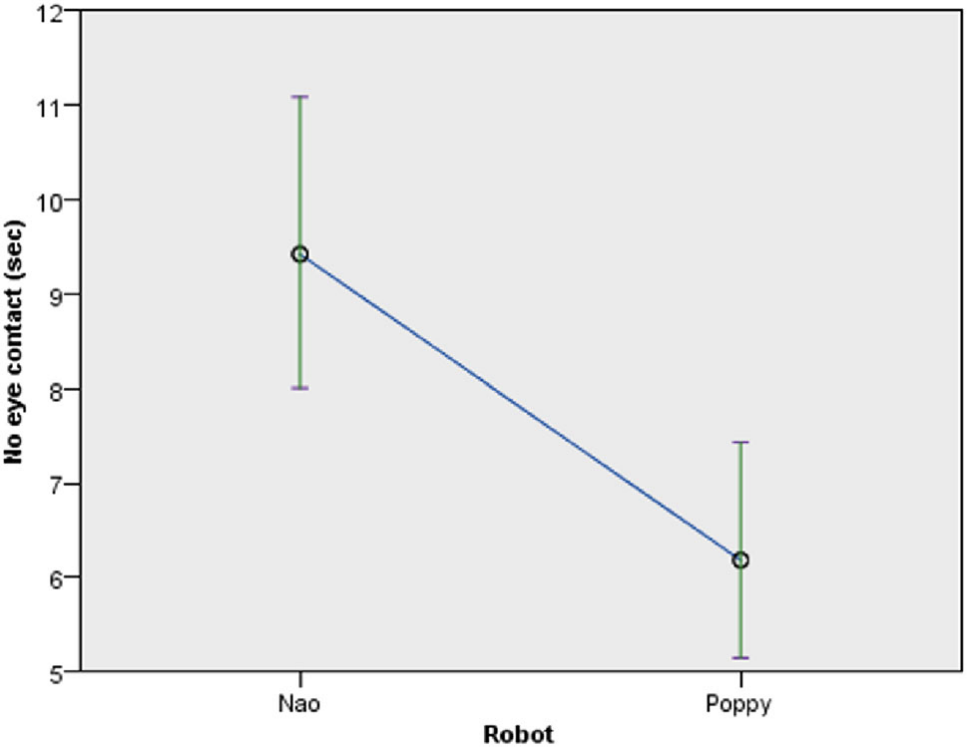
No eye contact by robot type

Engagement, as measured by the ratio of the participants’ “no eye-contact time” to trials total time, was significantly affected by the mode and timing of feedback, as well as the robot type. Considering the details of the eye-contact time ratio with respect to mode of feedback, participants who experienced visual and audio feedback were more engaged than participants who experienced audio feedback ($F(1,60) = 4.622$, *p* = 0.036) (Fig. 12 right). The ratio of participants who experienced audio and visual feedback (*M* = 0.036, *SD* = 0.06) was lower than participants with audio feedback (*M* = 0.403, *SD* = 0.0763) (confirming *H6a*). With respect to timing of feedback ($F(1,60) = 23.157$, *p* < 0.001) (Fig. 12 left), participants who experienced continuous feedback were more engaged than participants who experienced discrete feedback. The ratio for participants with continuous feedback (*M* = 0.03, *SD* = 0.048) was lower compared to participants with discrete feedback (*M* = 0.0483, *SD* = 0.865) (confirming *H6b*). With respect to robot type ($F(1,61) = 35.257$, *p* < 0.001) (Fig. 13), participants who interacted with Poppy robot were more engaged than participants who interacted with Nao robot. The ratio of the participants’ “no eye-contact time” to trials total time, with the Poppy robot (*M* = 0.029, *SD* = 0.0492) was lower than participants who used the Nao robot (*M* = 0.463, *SD* = 0.08) (confirming *H5*).

Regarding the subjective assessment of engagement through the questionnaires, the majority of the users indicated that they were engaged in the activity throughout the session. In terms of the feedback mode, all the participants in the audio and visual feedback group indicated they were engaged with the system while 93.5% of the participants who experienced audio feedback reported that they were engaged with the system (in line with *H6a*). The timing of the feedback and the robot type did not influence the engagement. The engagement was significantly affected by the users’ perceived ease of use (comfort $F(1,57) = 17.603$, *p* < 0.001, understanding $F(1,57) = 153.335$, *p* < 0.001))(confirming *H10*). Engagement was also significantly affected by the perceived usefulness ($F(2,60) = 7.069$, *p* = 0.002)(confirming *H9*).

Most of the participants indicated in the questionnaire that they trusted the robot. With respect to the feedback timing, most participants in the continuous feedback group (88.9%) indicated their trust compared to participants who experienced discrete feedback (71.4%) (in line with *H6b*) . With respect to the robot type, more participants who trained with Poppy (84.4%) trusted the robot than participants who trained with Nao (78.1%) (in line with *H5*). There was only a slight difference in the feedback mode preference regarding trust - 78.8% preferred the audio and visual while 83.9% preferred audio (contrary to *H6a*). Based on the subjective assessment, the trust was significantly affected by the understanding of the system ($F(1,56) = 9.67$, *p* = 0.003) (confirming *H10*) and by the perceived usefulness (*F*(2, 60) = 4.725, *p* = 0.012) (confirming *H9*).

Majority of the participants also indicated in the questionnaire that they were satisfied by the robots’ performance (with remaining 6/64 unsatisfied and 5/64 neutral). With respect to the feedback mode, more of the participants in the audio feedback group (90.3%) reported higher satisfaction from the robot compared to participants who experienced audio and visual feedback (81.8%) (contrary to *H6a*). With respect to the feedback timing, more of the participants in the continuous feedback group (94.4%) reported higher satisfaction compared to participants who experienced the discrete feedback (75%) (in line with *H6b*). The satisfaction of users who interacted with the Poppy robot (90.6%) was higher than the users who interacted with the Nao robot (81.3%) (in line with *H5*). Based on the subjective assessment, the satisfaction of the users was significantly affected by the users perceived usefulness (*F*(2, 60) = 4.911, *p* = 0.011) (confirming *H9*).

Regarding enjoyment, most of the participants also indicated in the questionnaire that they enjoyed the activity. In terms of the timing of the feedback, more of the participants in the continuous feedback group (94.4%) indicated their enjoyment compared to participants who experienced discrete feedback (75%) (in line with *H6b*). In terms of robot type, there was only a slight difference in the participants’ reported enjoyment while interacting with the robots—84.4% preferred Poppy while 78.1% preferred Nao (in line with *H5*). In terms of the feedback modes, audio and visual feedback was preferred (81.8%) over only audio feedback (80.6%) (in line with *H6a*). The enjoyment from the system was also significantly affected by the users perceived usefulness (*F*(2, 60) = 8.106, *p* = 0.001) (confirming *H9*). The results revealed that 97.8% of the participants would be willing to train with the robot in the future because it added value to them and they enjoyed the activity.

**Fig. 14:**
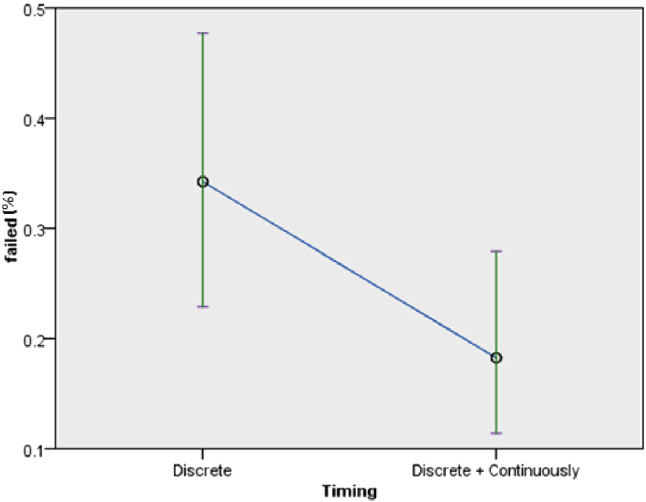
Success rate based on timing of feedback

**Fig. 15:**
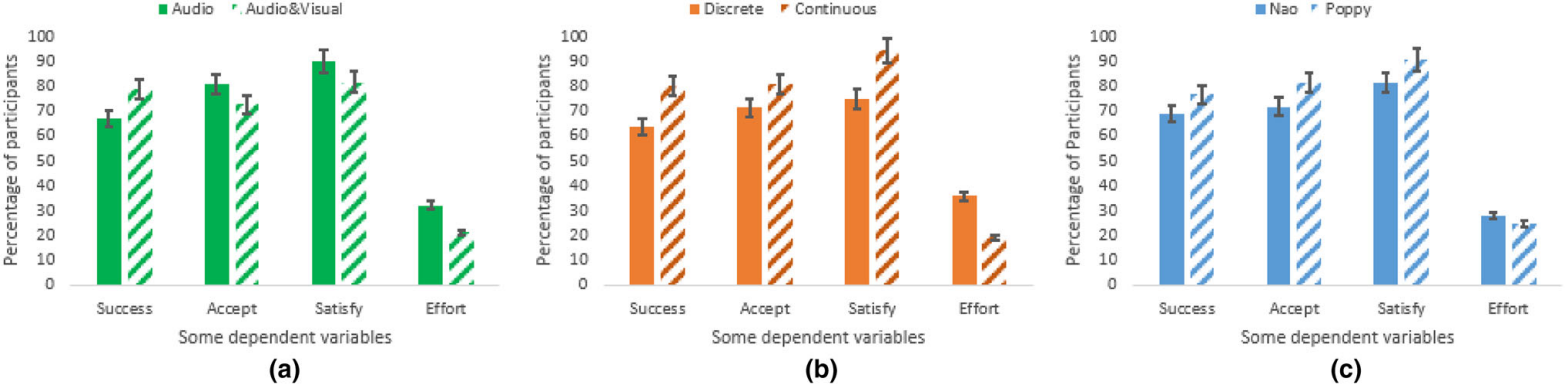
Overall participants’ assessment regarding influence of: **a** feedback mode, **b** feedback timing, **c** robot type. Success = Success rate, Accept = Acceptance, Satisfy = Satisfaction

The success rate of the participants was significantly affected by the timing of feedback, marginally affected by the robot type but not affected by the mode of feedback. The overall success rate was 73% (taking all the trials into consideration). With respect to the timing of feedback ($F(1,377) = 4.485$, *p* = 0.035) (Fig. 14), the success rate of participants with continuous feedback was 80% while that of participants with discrete feedback was 63.9% (confirming *H6b*). With respect to the robot type (*F*(1, 379) = 3.694, *p* = 0.055), 76.6% of the participants succeeded better with the Poppy robot as compared to the session with the Nao robot (which resulted in 69% success rate) (confirming *H5*). With respect to the feedback mode ($F(1,377) = 2.032$, *p* = 0.155), participants who experienced audio and visual feedback had a success rate of 78.8% while the success rate decreased to 67% for participants who experienced only audio feedback (in line with *H6a*).

### Intention to Use

Most of the participants indicated in the questionnaires their intention to use the system. With respect to the mode of feedback, more participants in the audio feedback group (80.6%) expressed their intention to use the system compared to participants in the audio and visual feedback group (72.7%) (contrary to *H8a*). With respect to the timing of feedback, a higher percentage of the participants in the continuous feedback group (80.6%) expressed their intention to use the system compared to participants in the discrete feedback group (71.4%) (in line with *H8b*). With respect to the robot type, a higher percentage of participants who trained with Poppy robot (81.3%), expressed their intention to use the system compared to participants who trained with Nao robot (71.9%) (in line with *H7*). The behavioral intention to use the robot was significantly affected by the users’ enjoyment (*F*(2, 53) = 7.421, *p* = 0.001). Analysis of results revealed that 92.3% of the participants who enjoyed the activity would like to exercise with the robot in the future (confirming *H11*).

## Discussion

### Overall Assessment of the Participants and Interaction

The TAP and NARS results provided some general background about the participants’ willingness to explore new technology. This is depicted in the results where more than 95% of the participants had at least a medium propensity to adopt new technology. Also, the fact that about half of the participants had a neutral attitude towards robots provided a fair basis for comparison of the main variables in the participants’ interaction with the robot.

In terms of the main variables assessed, the timing of feedback and the robot type influenced most of the interaction variables (confirming *H3b*, *H5*, *H6b*, and in line with *H1b*, *H4*, *H7*, *H8b*). The mode of feedback influenced only some of the interaction parameters (confirming *H3a* and *H6a*). A depiction of the overall assessment for selected variables under ease of use, attitude, intention to use is presented in Fig. 15. The effect of each of these variables on the interaction is discussed below in detail.

### Mode of Feedback

Combining visual and audio feedback, especially in the system that included the Poppy robot, helped the users to understand the instructions of the robot better [62]. It reduced their reaction time and increased the persistence of training. We believe that visual feedback combined with audio will help the older adults to understand the system even if they have hearing limitations and as a result do not hear the audio feedback.

The positive effect the combined audio and visual feedback had on the ease of use and positive engagement by the system aligned with the findings of another study [37] that stated that verbal feedback supported by another feedback modality provides more positive outcome.

Existing studies recommend that feedback should be adequate and informative [63]. To avoid overloading the user with information, the information content should be minimal [64]. In our study the information provided was just related to the number of times the user should perform the exercises. At the end of the exercise, the robot commended the user whenever the user succeeded and also asked if the user liked to do some more exercises.

Based on the assessment of the ease of use, attitude and intention to use, we recommend the audio and visual as the mode of feedback which provided higher success rate.

### Timing of Feedback

The continuous feedback seemed to have positively influenced the success rate, engagement and the enjoyment of the system. Trust of the participants also seemed to build with the continuous feedback because it seemed to keep the users better informed during the exercise session and not only at the end.

Continuous feedback with counting helped part of the users keep track of the number of right steps and repetitions they had made. Some of the participants with discrete feedback experienced uncertainties regarding their performances in the exercise; they were unsure if they made the correct movement and they were not confident about it. This correlates with the existing literature which states that continuous feedback keeps the users constantly aware of the state of the interaction at every point in time [39]. However, some noted that they preferred the system without counting feedback. We assume that for those people, the robot’s feedback in ’counting’ form during the exercise, could have been excessive, as observed in some studies that too much information could cause confusion for the users [64]. However, the results in the current study are in line with the literature [40] where continuous feedback was found to provide better understanding, enjoyment, and trust of the system by the users. Continuous feedback was also observed to improve the flow of interaction.

### Robot Type

The Poppy robot engaged the users more than the Nao robot. We think that the facial expression on the screen of Poppy had some influence on the positive perception of the users as noted in previous literature [35]. Such facial expressions seem to make the robots behavior better understandable and more attractive corresponding to previous research [25]. Another reason might be the more mechanical look of the Poppy versus the humanoid toy look of the Nao robot.

The users also seemed to trust the Poppy robot more than the Nao robot. A reason could be due to the observable mechanical parts and wires on the Poppy robot which may have given it more of the semblance of a robot compared to the Nao robot.

### Limitations

Some of the limitations of this research were related to:

- Variability in the participants—there is high variability in the personality differences of older adults, and this influences their preferences.
- Novelty effect—it was the first time that the users interacted with a robotic coach and their assessments may change over an extended period of interaction.
- More assessments with the gaze monitoring system—this could be taken to improve the accuracy of the gaze duration but was not taken in this study in order to avoid burdening the participants.
- Cognitive, hearing and vision condition of the participants—a standard assessment of these measures were not taken. It is noteworthy that cognitive, hearing or visual impairments can affect the interaction of the users with the robot and influence the results and therefore should be considered in future studies.

## Conclusions and Future Work

The robotic system was designed to motivate older adults to engage more in physical exercises. Continuous feedback provided in combined audio and visual mode, using a mechanical-like robot increased ease of use for the older adults. These resulted in a better positive attitude and intention to use. However, the combined audio and visual feedback and robot type did not influence the perception of the older adults regarding the usefulness of the robot and their intention to use.

Participants used the system without problems, and understood the interaction with the robot despite the fact that most of them were novice users. The eagerness of the participants to train with a robotic coach revealed a potential of the system to be used as a personal physical trainer for the older adults.

Future work should include additional exercises for the robotic system. Aerobics and lower body exercises could also be included as complementary exercises for more holistic physical training sessions for the older adults. Adding difficulty levels for the users that will be adaptive to the users’ performance and level is also recommended for further study.

Incorporating an emotion recognition learning algorithm in the system has the potential to improve the system feedback. Such a learning algorithm can enable the system to classify the users emotion (bored, exhausted, fascinated, happy, etc.) and thus provide better feedback with recommendations for improved performance (more challenging exercises, extended training, games, etc.).

Incorporating an imitation learning algorithm to mirror the movements of the users can provide real-time assessment of the users’ performance. It could help the users see the kind of movements being made and guide them in correcting wrong movements. It could also enable the inclusion of additional tailored design exercises provided by physical therapists or personal trainers.

Ongoing research is aimed at performing a long-term study with more participants (with different older adults age groups and gender) that will assess a wider change of preferences and explore the differences in the reaction and preferences of the older adults over a longer period. Additionally, we are developing a more holistic training system by adding cognitive training and relaxation exercises which have proven necessary along the COVID-19 pandemic in which many older adults were quarantined at home for extended periods.

On the whole, the robotic system developed and evaluated in this study reveals the potential of improving the physical training experience of older adults with a robotic coach and the importance of including best-fit feedback to improve its usability and acceptance.

## Footnotes

1 https://www.care-o-bot.de/en/research.html.

2 https://www.robotcaresystems.com/.

3 https://www.intuitionrobotics.com/elliq/.

4 https://kompairobotics.com/why-robots-can-help-frail-people/.

5 https://www.ald.softbankrobotics.com/en.

6 https://www.nia.nih.gov/health/exercise-physical-activity.

7 https://www.cdi-negev.com/project/the-healthy-aging-simulation-center/.

8 https://buy.garmin.com/en-US/US/p/529988.
